# Evaluation of Flexible Central Buckles on Short Suspenders’ Corrosion Fatigue Degradation on a Suspension Bridge under Traffic Load

**DOI:** 10.3390/ma16010290

**Published:** 2022-12-28

**Authors:** Yue Zhao, Xuelian Guo, Botong Su, Yamin Sun, Xiaolong Li

**Affiliations:** 1 School of Civil Engineering and Architecture, Xi’an University of Technology, Xi’an 710048, China; 2 School of Highway, Chang’an University, Xi’an 710064, China; 3 School of Architecture and Civil Engineering, Xi’an University of Science and Technology, Xi’an 710054, China

**Keywords:** suspender, high-strength steel wire, corrosion fatigue, flexible central buckle, bending stress

## Abstract

Suspenders are the crucial load-bearing components of long-span suspension bridges, and are sensitive to the repetitive vibrations caused by traffic load. The degradation of suspender steel wire is a typical corrosion fatigue process. Although the high-strength steel wire is protected by a coating and protection system, the suspender is still a fragile component that needs to be replaced many times in the service life of the bridge. Flexible central buckles, which may improve the wind resistance of bridges, are used as a vibration control measure in suspension bridges and also have an influence on the corrosion fatigue life of suspenders under traffic load. This study established a corrosion fatigue degradation model of high-strength steel wire based on the Forman crack development model and explored the influence of flexible central buckles on the corrosion fatigue life of suspenders under traffic flow. The fatigue life of short suspenders without buckles and those with different numbers of buckles was analyzed. The results indicate that the bending stress of short suspenders is remarkably greater than that of long suspenders, whereas the corrosion fatigue life of steel wires is lower due to the large bending stress. Bending stress is the crucial factor affecting the corrosion fatigue life of steel wires. Without flexible central buckles, short suspenders may have fatigue lives lower than the design value. The utilization of flexible central buckles can reduce the peak value and equivalent stress of bending stress, and the improved stress state of the short suspender considerably extends the corrosion fatigue life of steel wires under traffic flow. However, when the number of central buckles exceeds two, the increase in number does not improve the service life of steel wire.

## 1. Introduction

With the rapid development of highway transportation, long-span suspension bridges are constructed across mountains, valleys, and rivers for their good mechanical characteristics and excellent spanning performance. The construction of early large-span suspension bridges was limited by experience and technology, and structural vibration control measures were relatively lacking, leading to obvious vibration responses under external load. The longitudinal vibration displacement of structures caused by external load may lead to the fatigue of expansion joints and other ancillary components. Traffic load has been proven to be one of the main reasons causing the longitudinal vibration displacement of structures. Such vibration may cause the fatigue of expansion joints and other ancillary components [1,2]. Suspenders are key load-bearing components of suspension bridges, of which the degradation is the result of the comprehensive action of corrosion and fatigue, and the corrosion accelerates the generation of fatigue cracks. The corrosion degradation of components seriously affects the reliability of bridge operation [3]. The propagation of fatigue cracks is easily affected by vibrations under traffic load; thus, it has been an important research goal to evaluate the stress and service life of short suspenders. Flexible central buckles were set up in the midspan for long-span suspension bridges recently to enhance wind resistance performance with low cost and construction convenience, but the studies on them were limited and mainly focused on wind resistance and the vibration characteristics of the structure itself. Actually, flexible central buckles also play a contributing role to the suspenders’ response under traffic flow that may reduce the fatigue degradation of suspenders, but the influence mechanism on the structural vibration under traffic load remains unclear. Thus, the control effect of flexible central cables should be investigated to optimize the designation of the flexible central buckle.

Corrosion fatigue is the phenomenon of crack formation and propagation under the interaction of alternating load and a corrosive medium that leads to a reduction in fatigue resistance [4]. Scholars have studied the corrosion fatigue degradation process of high-strength steel wires in bridge engineering. The surface of the suspender steel wire is provided with a coating to enhance the corrosion resistance. The damage to the coating’s passive film is accompanied by pitting corrosion. Roffey indicates that the pit corrosion of the steel wire develops into vertical cracks inside, resulting in the decline of the bearing capacity of the steel wire based on the inspection results of the Fourth Highway Bridge in Scotland [5]. Qiao Yan divided the corrosion fatigue process of steel wire into three stages—coating corrosion, corrosion pit development, and crack development—and gave a calculation method for the development time of each stage [6]. Valor proposed a random model for pitting distribution simulation which uses a non-uniform Poisson process to simulate the generation of pits and verified it with experiments [7]. Nakamura investigated the corrosion of steel wires in different environments for fatigue loading. The results show that the fatigue life of steel wire in a corrosive environment decreases significantly [8]. Suzumura studied the effects of reagent concentration, ambient temperature, and humidity on the corrosion rate of galvanized steel wire through experiments, and gave the loss rate of zinc coating on galvanized steel wire [9]. Although the durability of galvanized steel wire has been significantly improved, it still does not meet the engineering requirements. The corrosion resistance of the coating can be achieved by improving the properties of the coating, such as improving the adhesion and porosity and adding elements that can form a passive film; it is proven that the corrosion resistance can be improved by the oxidation of the Al element [10]. In recent years, Galfan steel wires have been gradually widely used. The evaluation method of steel wire has been well developed, but the existing models mainly use the Paris criterion to calculate the crack growth rate; the influence of the average load factor and the difference in crack growth rate caused by the change in traffic flow intensity are not considered. There is a deviation when using the parameters under the same stress ratio to calculate the crack growth life. 

Furthermore, the axial stress and bending stress fluctuations caused by relative displacements between the girder and cables easily damage the short suspenders along with fatigue degradation [11,12]. To reduce the fatigue damage of short suspenders, appropriate vibration control facilities are utilized to control bridge vibration [13,14]. The central buckle is a vibration control measure for long-span suspension bridges, which includes a flexible central buckle and a rigid central buckle. Previous research focuses on the influence of rigid central buckles on the dynamic characteristics of bridges [15]. Wang analyzed the influence of rigid central buckles on the wind-induced buffeting response of long-span suspension bridges and pointed out that rigid central buckles can suppress buffeting vibration [16]. Wang investigated the working and mechanical characteristics of the rigid central buckle of the Runyang Yangtze River Bridge under vehicle load based on measured results and finite element modeling [17]. Liu investigated the effects of central clamps in the midspan (i.e., rigid central buckle) on the fatigue life of short suspenders, and the results revealed that short suspenders were more prone to fatigue than others because of large bending stress, and central clamps can effectively improve their lifespan [18]. In addition to a rigid central buckle, a flexible central buckle cable was set up in the midspan to enhance wind resistance performance. Wang studied the influence of flexible central buckles on the displacement of stiffening girders. The results showed that the flexible central buckle remarkably reduces the longitudinal amplitude of the stiffening girder and increases its vibration frequency [19]. The influence of flexible buckles on structural vibration under random traffic flow remains unclear. The control effect of flexible buckles under random traffic flow should be studied.

Traffic flow is an important vibration source in the suspender stress response. Suspension bridges are a flexible system and structural deformation is evident under the action of traffic flow, which varies with traffic density. Characteristics such as traffic flow parameters, vehicle type, and vehicle weight generally have random distribution [20,21]. The load effect of traffic flow can be well considered by a macro traffic flow simulation method [22,23]. Thus, in this study, the influence of flexible central buckles on the stress response and corrosion fatigue life of suspenders under traffic flow were analyzed by numerical modeling. First, the corrosion fatigue of high-strength steel wire based on the Forman criterion was established. Then, the response of suspenders with flexible central buckles was calculated with consideration of the load effect of traffic flow at different levels. Finally, the fatigue life of suspender steel wires and the influence of flexible central buckles were evaluated. This research can provide a reference for the design and maintenance of long-span suspension bridges.

## 2. Prototype Bridge

### 2.1. Bridge Information

**Figure 1:**
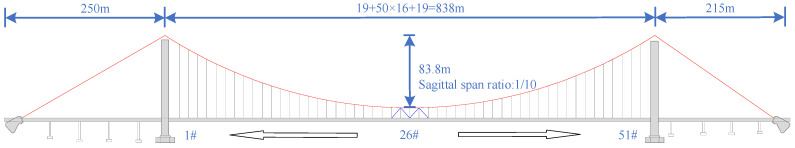
Layout of the prototype bridge.

**Figure 2:**
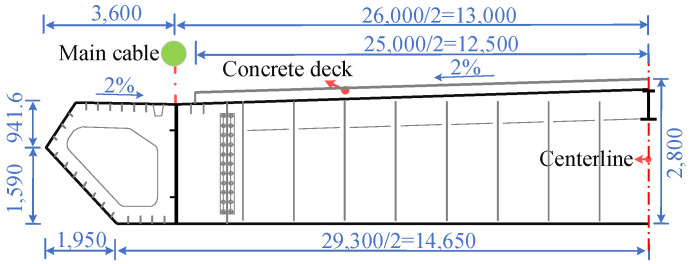
Half-section of the stiffening girder (mm).

This study takes the Zhixi Yangtze River Bridge as the research object. The bridge is a single-span steel–concrete composite girder suspension bridge. The section layout is shown in Figure 1. The span of the main cable is arranged as 250 + 838 + 215 m, and the sagittal span ratio of the midspan main cable is 1/10. The standard distance between the adjacent lifting points of stiffening girders is 16 m, and the suspender adopts a φ5.0 mm galvanized aluminum alloy (i.e., Galfan coating) high-strength steel wire. To improve the vibration resistance of the bridge, two flexible central buckles are set near both sides of the middle span of each main cable to form a cable–beam connection. The entire bridge has a total of eight central buckles. The stiffening girder adopts a steel–concrete composite structure in which the steel beam is combined with the concrete deck through shear nails. The half section of the stiffening girder is shown in Figure 2. The full width of the stiffening girder is 33.2 m, the center height is 2.8 m, and the central transverse spacing of the two main cables in the midspan is 26.0 m. The small longitudinal beams are arranged longitudinally at the center line of the girder and the top surface is flush with the top surface of the steel beam. The bridge deck is reinforced concrete with a full width of 25.0 m and a thickness of 0.22 m.

**Figure 3:**
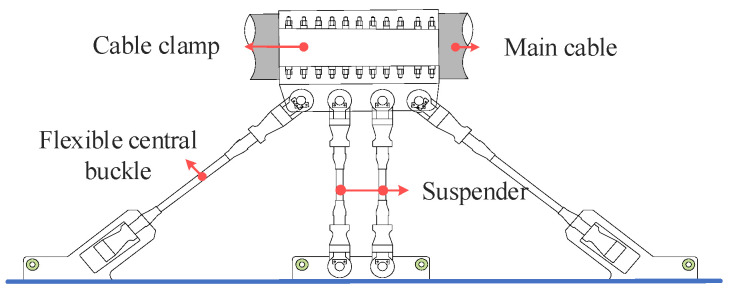
Flexible central buckle.

As a common vibration control measure, central buckles are used to improve the vibration response of suspension bridges. These buckles are generally installed in the middle span; examples include the Runyang Yangtze River Bridge and the Sidu River Bridge, in which the rigid central buckle is installed in the middle span. Existing research indicates that the rigid central buckle can improve the structure frequency and reduce the longitudinal displacement response of the girder [24]. In the Zhixi Yangtze River Bridge, flexible central buckles that differ from traditional rigid central buckles are set in the middle of the main span of each main cable to coordinate with short suspenders, as shown in Figure 3. The flexible central buckle is composed of an inclined cable connected to a short suspender, forming a cable–girder connection to control the vibration response of the structure.

### 2.2. Finite Element (FE) Model

**Figure 4:**
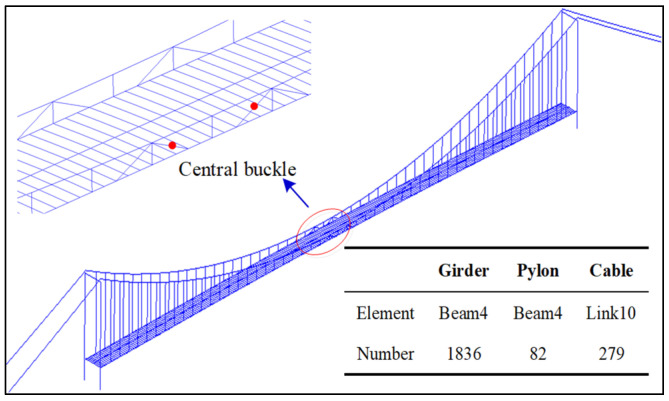
Bridge FE model.

To simulate the structural characteristics, a three-girder model of a prototype bridge was established using ANSYS 18.0. The FE model is shown in Figure 4. The stiffening girder of the bridge is a steel-composite girder with an open section, and the longitudinal beams on both sides are the main bearing structures of the stiffening girder. Thus, the BEAM4 element was used to simulate the main stringer, small stringer, steel beam, and main tower. The LINK10 element was used to simulate the cable components. A total of 1836 BEAM4 elements for the girder, 82 BEAM4 elements for the pylon, and 279 LINK10 elements for the main cable and suspender were found. The bridge deck pavement contributes minimally to the stiffness of the stiffening girder; thus, only its mass was considered, and the stress stiffening of the LINK element was conducted in accordance with the measured cable force. 

**Figure 5:**
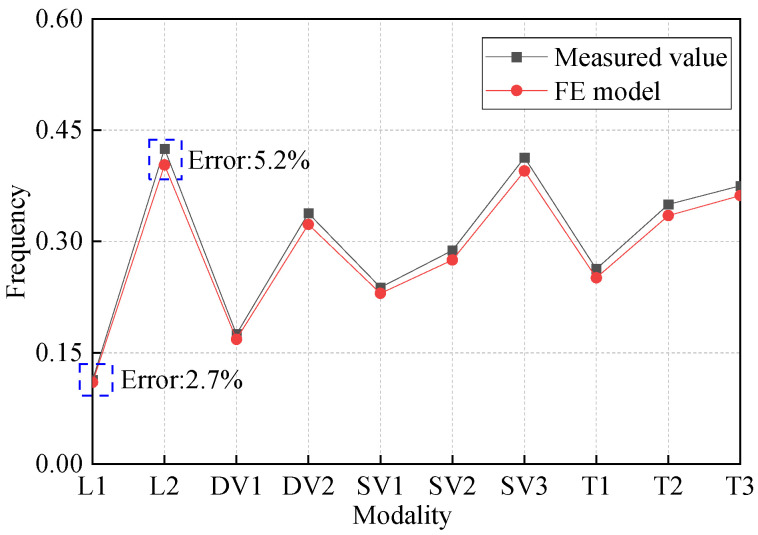
Comparison of structure frequency (L is lateral mode; DV is dissymmetry vertical mode; SV is symmetrical vertical mode; T is torsional mode).

The theoretical material properties and cable force vary from the actual state of the structure; thus, the FE model was modified according to the measured material properties and cable force in construction. Then, the structure frequency was calculated by the modal analysis module of ANSYS software, and the Block Lanczos feature solver based on the Lanczos algorithm was used in modal analysis. When calculating the natural frequencies of a certain range contained in the eigenvalue spectrum of a system, the Block Lanczos method is particularly effective for extracting modes. The frequencies of the FE model were compared with measured structure frequency to validate the FE model. The research team undertook the monitoring of the structural state during the bridge’s construction. After construction, the actual vibration mode and frequency of the bridge were measured through the modal test analysis system. Figure 5 shows the modal analysis results and the test results; the modes of vibration are consistent with the test results. The first-order frequency L1 is 0.113 hz and smaller than other bridge types, which is determined by the flexibility characteristics of the suspension bridge. The error of L1 is 2.7%, and the maximum error is 5.2% in L2. The errors are within the acceptable range, which preliminarily proves the simulation effect of the FE model.

**Figure 6:**
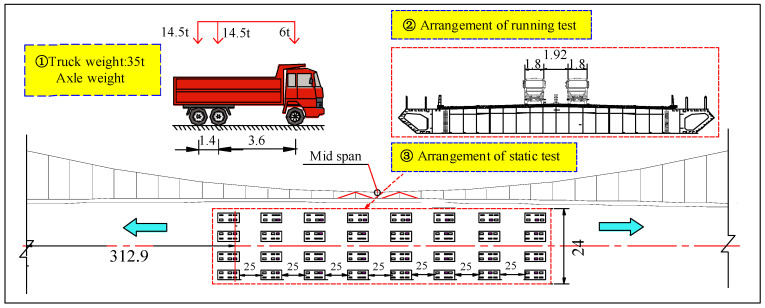
Load case of bridge static and running tests. (unit: m).

**Figure 7:**
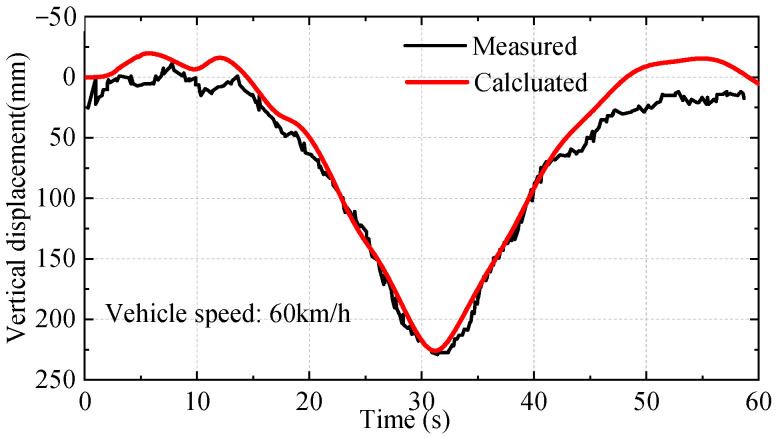
Comparison of midspan dynamic strain under running test.

Besides the modal test, the vehicle loading experiment was conducted to test its deformation performance under external load. Figure 6 shows the layout of the static and running tests of the bridge. The loading vehicle is a 35 t three-axle truck. The static test has four loading trucks in each row and eight rows in total. A comparison of the maximum girder vertical deflection of the static test is given in Table 1. The computing value and measured value are close, and the error is within 3%. The running test condition is that two 35 t loading vehicles drove through the bridge at a constant speed of 60 km/h, and the vertical dynamic deflection of the main girder in 1/2 L is measured. A comparison between the measured results and the FE model is shown in Figure 7; the results are in good agreement. The model can reflect the dynamic response of the bridge and satisfy the requirements of subsequent analysis under traffic flow.

## 3. Corrosion Fatigue of Suspender Steel Wire

### 3.1. Corrosion Fatigue Mechanism

**Figure 8:**
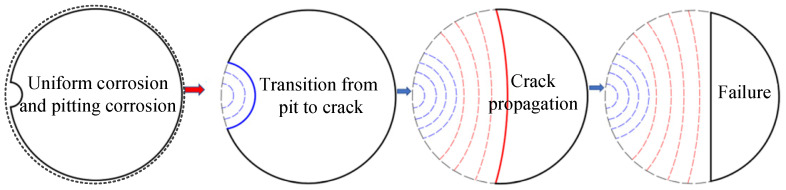
Degradation of suspender steel wires.

The stress of long-span bridge suspenders is caused mostly by dead load; thus, the amplitude of stress change caused by vehicle load and other live loads is relatively small and is far lower than the fatigue limit of steel wire. Therefore, the degradation process is a typical corrosion fatigue process; that is, the corrosion defects on the steel wire surface develop into initial crack damage. The entire steel wire degradation process can be divided into stages of the development of corrosion and crack propagation, as shown in Figure 8. The tiny corrosion defects on the steel wire surface become the crack initiation site. When the corrosion defects transform into cracks, they continue to develop until destroyed under the action of load cycles. This degradation process can be simulated by the corrosion fatigue theory.

### 3.2. Uniform Corrosion and Pitting Corrosion

The corrosion of steel wire includes uniform corrosion and pitting corrosion. Uniform corrosion describes the degree of average corrosion of the steel wire surface, which directly causes the reduction in the diameter of the steel wire, and the extent of diameter reduction is assumed to stay unchanged along the steel wire length [25]. The steel wire parameters adopted in this study are shown in Table 2. The surface of high-strength steel wire is usually protected by a coating for corrosion resistance. In the prototype bridge, the suspender consists of Galfan-coated steel wires. The Galfan coating should not be less than 300 g/m^2^ due to the specification of bridge designation [26]. According to the survey of relevant cable manufacturers, the coating quality is usually controlled within 350 g/m^2^. Thus, the depth of a Zn-Al alloy coating can be calculated according to its density (6.58 g/cm^3^) and ranges from about 29 μm to 34 μm. 

The uniform corrosion of high-strength steel wire undergoes a two-stage corrosion process; that is, the corrosion of the coating and the corrosion of the steel wire substrate. The corrosion rate can be described as Equation (1).

(1) $$a_{u}\left(t\right)=\left\{\begin{array}{c} d_{c}\left(t\right)\;t\leq t_{c}\\ d_{s}\left(t\right)+d_{c}\left(t\right)\;t\geq t_{c} \end{array}\right.$$

where $a_{u}\left(t\right)$ is the depth of uniform corrosion, *d*_c_ is the corrosion depth of the zinc–aluminum alloy coating, *d*_s_ is the corrosion depth of the steel wire, *t* is corrosion time, and *t*_c_ is the time when the coating is totally corroded.

According to the preliminary work of the research team, the corrosion process of Galfan steel wire is measured by an accelerated corrosion test and can be simulated by parabola distribution as Equation (2) [25]. The corrosion rate decreases gradually. The oxidation products of aluminum in the coating form a passive film, which slows down the corrosion rate.

(2) $$a_{u}\left(t\right)=0.04431t-0.000014t^{2}$$

where *t* is corrosion time.

In service conditions, the corrosion rate of Galfan coating is significantly different because of the exposure environment. As is well known, field exposure tests are difficult to conduct due to the high cost of time, and it is also difficult to find exactly matched field exposure test results. Thus, the time conversion scale was determined by the field exposure test of Galfan coating by Aoki and Katayama, in which a hot-dipped Galfan-coated steel plate with a 25 μm coating was investigated [27,28]. Assuming that the influence of coating thickness and surface shape on the corrosion rate is negligible, and the test results are applicable to the conversion time scale, it is suggested that 1 h of the accelerated corrosion test corresponds to 0.033~0.052 years in a rural environment, 0.018~0.024 years in an industrial environment, 0.019~0.028 years in a marine environment, and 0.014~0.022 years in a severe marine environment.

When the metal material surface has a passive or protective film, the pitting pit on the substrate surface appears after the protective layer is consumed, greatly affecting the characteristics of the steel wire. Pitting corrosion occurs randomly, accompanied by uniform corrosion [29]. Given the stress concentration effect, pitting corrosion is the site where steel wire fatigue fracture may occur. The pitting pit with the largest depth determines the working state of the steel wire; thus, the pitting pit with the largest depth is the key analysis point in corrosion fatigue analysis. Pitting pit depth can be calculated by uniform corrosion depth and pitting coefficient.

(3) $$\wedge \left(t\right)=a_{p}\left(t\right)/a_{u}\left(t\right)$$

where $a_{p}\left(t\right)$ and $a_{u}\left(t\right)$ denote the depth of pitting corrosion and uniform corrosion.

The distribution of the maximum pitting coefficient conforms $\wedge \left(t\right)$ to the Gumbel distribution [7], which can be expressed as

(4) $$F\left(\wedge \left(t\right)\right)=\exp\{-\exp[-\frac{\left(\wedge \left(t\right)-\beta_{0}\right)}{\alpha_{0}}]\}$$

where $F\left(\wedge \left(t\right)\right)$ is the cumulative probability density function; $\wedge \left(t\right)$ is the maximum pitting coefficient; and $\alpha_{0}$ and $\beta_{0}$ are the distribution parameters.

Then, the distribution parameter of any wires with different lengths and diameters can be calculated by Equation (5):

(5) $$\beta_{k}=\beta_{0}+\frac{1}{\alpha_{0}}\ln(\frac{A_{k}}{A_{0}}),\alpha_{k}=\alpha_{0}$$

where $A_{k}$ is the surface area of the analysis target, and $A_{0}$ is the surface area of the wire with a 125 mm length and 8 mm diameter.

### 3.3. Corrosion Fatigue Crack

Stress concentration happens due to the shape characteristics of the corrosion pit. As the depth of the corrosion pit increases, a crack will occur when the stress intensity reaches a critical value. The transition process from pitting to cracking can be determined by two methods: (1) the growth rate of the fatigue crack exceeding that of the corrosion pit and (2) the stress intensity factor of the corrosion pit reaching the critical threshold of fatigue crack propagation. This study adopts the former method. The steel wire crack dominates when the development speed of the pitting pit depth exceeds that of the crack.

Corrosion cracks expand until failure under the stress cycle caused by an operating live load. The Forman formula is used to analyze the growth rate of a metal corrosion fatigue crack, as shown in Equation (6).

(6) $$\frac{d_{a}}{d_{N}}=C{\left(\Delta K\right)}^{m}/\left[K_{c}\left(1-R\right)-\Delta K\right]$$

where $\frac{d_{a}}{d_{N}}$ is the growth rate of the crack, *a* is the depth of the crack, *C* and *m* are the parameters of the Paris criterion [30], $K_{c}$ is the fracture toughness of the material, ΔK is the stress intensity factor range, and *R* is the stress ratio of alternating load.

The stress intensity factor ΔK is given by Forman, as follows:

(7) $$\Delta K=F_{a}\left(\frac{a}{b}\right)\Delta \sigma_{a}\sqrt{\pi a}+F_{b}\left(\frac{a}{b}\right)\Delta \sigma_{b}\sqrt{\pi a}$$

where $F_{a}\left(\frac{a}{b}\right)$ denotes a coefficient related to axial stress, $F_{b}\left(\frac{a}{b}\right)$ denotes a coefficient related to bending stress, *a* is the crack depth, *b* is the diameter of the steel wire, $\Delta \sigma_{a}$ is the equivalent axial stress amplitude, and $\Delta \sigma_{b}$ is the equivalent axial stress amplitude.

$F\left(\frac{a}{b}\right)$ is calculated by Equation (8) [31].

(8) $$\left\{\begin{array}{c} F_{a}\left(\frac{a}{b}\right)=0.92\cdot \frac{2}{\pi }\cdot \sqrt{\frac{2b}{\pi a}\cdot tan\frac{\pi a}{2b}}\cdot \frac{0.752+1.286(\frac{a}{b})+0.37{\left(1-sin\frac{\pi a}{2b}\right)}^{3}}{cos\frac{\pi a}{2b}}\\ F_{b}\left(\frac{a}{b}\right)=0.92\cdot \frac{2}{\pi }\cdot \sqrt{\frac{2b}{\pi a}\cdot tan\frac{\pi a}{2b}}\cdot \frac{0.923+0.199{\left(1-sin\frac{\pi a}{2b}\right)}^{4}}{cos\frac{\pi a}{2b}} \end{array}\right.$$

where *a* is the crack depth, and *b* is the diameter of the steel wire.

To consider the effect of daily traffic flow on the structure comprehensively, a crack depth development model is established on the basis of daily traffic flow operation according to the traffic load investigation, as shown in Equation (9).

(9) $$\left\{\begin{array}{c} a_{i}=\Delta a+a_{i-1}\\ \Delta a=C\sum e_{j}N_{j}{\left(\Delta K_{j}\right)}^{m}/\left[K_{c}\left(1-R\right)-\Delta K\right] \end{array}\right.$$

where $a_{i}$ is the depth of the crack at time *i*; Δa is the increment of the crack; $e_{j}$ is the operating time of traffic flow with different intensities; $\sum e_{j}=24$ h; and $\Delta K_{j}$ and $N_{j}$ are the stress intensity factor range and the number of cycles, respectively. Mayrbaurl pointed out that the critical relative crack depth conforms to the lognormal distribution with an average value of 0.390 and a coefficient of variation of 0.414. Based on the test, the maximum critical relative depth is 0.5, which is used as the judgment standard for steel wire failure.

## 4. Traffic-Induced Stress Responses of Suspenders

### 4.1. Vehicle Bridge System

**Figure 9:**
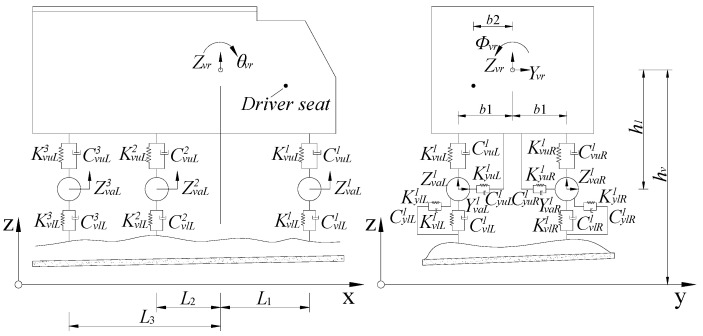
Dynamic model of a three-axle vehicle.

Vehicles can be classified into different types according to axle distance, axle number, vehicle load, etc. Vehicle subsystems are commonly simplified as a car body, wheels, a shock mitigation system, and a damping system. The corresponding dynamic models are established on the basis of the hypothesis that the mass of the damper and spring components are ignored. For example, a three-axle vehicle is shown in Figure 9 [32]. The longitudinal vibration of the vehicle is neglected for its few effects on the bridge; thus, the longitudinal degree of freedom is ignored in the analysis [33]. Thus, five degrees of freedom (vertical, horizontal, head nodding, side rolling, and head shaking) are considered for the integral vehicle. The vehicle dynamic models are also classified into five types, and the corresponding dynamic models are constructed.

Vehicle wheels always keep contact with the deck; the bridge deformation caused by an external load leads to the vibration response of the vehicle and bridge subsystems; the dynamic response is influenced by the overall total mass matrix, damping matrix, and the overall stiffness matrix of the subsystem; and the road surface roughness is the main excitation source. Therefore, the interaction force between the vehicle and bridge system is a function of the vehicle–bridge system’s motion state and road roughness, which can be analyzed in the established vehicle–bridge analysis system [34]. The road surface roughness is described by a power spectral density function, which can be generated through Fourier inversion [35].

(10) $$\left\{\begin{array}{c} F_{v}=F_{vi}\left(Z_{v},{\dot{Z}}_{v},{\ddot{Z}}_{v},Z_{b},{\dot{Z}}_{b},{\ddot{Z}}_{b},i\right)\\ F_{b}=F_{bi}\left(Z_{v},{\dot{Z}}_{v},{\ddot{Z}}_{v},Z_{b},{\dot{Z}}_{b},{\ddot{Z}}_{b},i\right) \end{array}\right.$$

where $Z_{v}$ denotes vehicle displacement, $Z_{b}$ denotes bridge displacement, and *i* denotes road surface roughness.

### 4.2. Traffic Load Simulation

**Figure 10:**
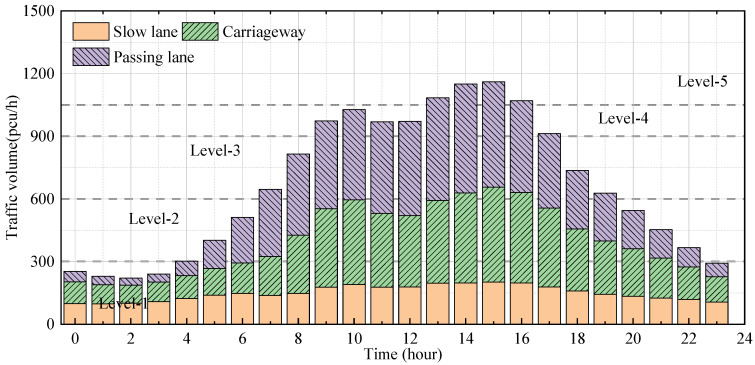
Average traffic volume from WIM data.

The vehicle load data monitored in a region is used to further evaluate the degradation process of suspenders under traffic load. The data were collected from the traffic load of a long-span bridge for one month by a weigh-in-motion (WIM) system. Figure 10 shows the hourly traffic volume results of the traffic flow, which are divided into five levels based on the range of traffic volume, including level 1 (<300 passenger car unit (pcu)/h), level 2 (300~600 pcu/h), level 3 (600~900 pcu/h), level 4 (900~1050 pcu/h) and level 5 (>1050 pcu/h). The error bar of the hourly traffic volume proves that the traffic volume is relatively stable. Although the standard deviation of peak traffic volume is larger than the trough period, the overall distribution is consistent, which does not affect the division of traffic intensity. The time proportions are 0.25, 0.21, 0.165, 0.21, and 0.165, respectively. The established random traffic flow simulation method is used to generate traffic flow loads of different strengths for loading [34], so as to obtain the impact of traffic flow level on the stress response of the suspenders.

## 5. Numerical Analysis

### 5.1. Analysis Conditions

As a tensioned component, flexible central buckles cannot support the vibration response of the midspan main beam or cable as the rigid central buckles, but they can still affect the overall response of the structure by changing the fastening force. The connection system formed by the central cable and the suspenders changes the distribution of the force of the suspender near the midspan under traffic load. Traffic load is the main inducement of the bridge vibration response. To study the improvement effect of central buckles on bridge vibration, this study analyzes the response of bridges under conditions such as no central buckle and settled flexible central buckles. The detailed analysis conditions are shown in Table 3. 

### 5.2. Evaluation Process

**Figure 11:**
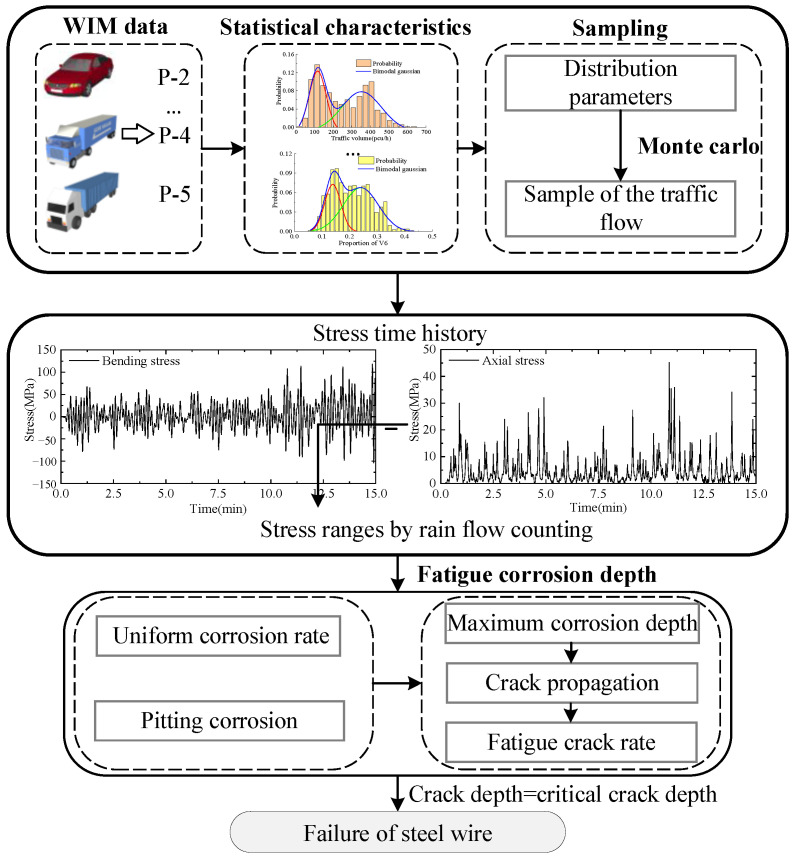
Simulation process of wire life.

The fatigue life of suspender wires can be predicted by the corrosion fatigue theory, and the detailed prediction process is shown in Figure 11. Considering the traffic density variation, the contribution of the corresponding stress cycle times to crack depth is calculated on the basis of the hourly occupancy rate of different traffic flows in one year, and the change law of crack depth and crack development rate with time is obtained. The process includes the following steps: (1) analyzing the characteristics of traffic flow parameters and generating random traffic flow samples on the basis of the WIM data; (2) taking traffic flow into the vehicle bridge coupling analysis system and obtaining the time history results of suspender stress; (3) simulating the uniform corrosion process of steel wire and generating random samples of pitting corrosion; (4) calculating crack propagation by integrating vehicle flow effects of different levels until failure.

### 5.3. Result Discussion

**Figure 12:**
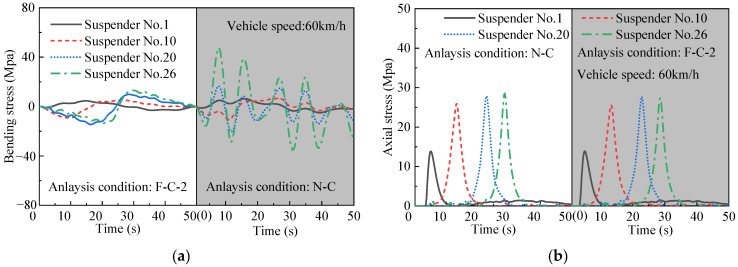
Comparison of suspender stress. (**a**) Bending stress; (**b**) axial stress.

The dynamic test under truck load in Figure 6 is used to analyze the dynamic response of the bridge structure with or without the central buckle. First, the suspender response of the running test is shown in Figure 12 to analyze the difference between suspenders. The suspender bears axial stress and bending stress due to the relative movement between the main cable and the stiffening beam. The bending stress of the suspender cannot be directly obtained by the LINK10 element. Thus, the Wyatt theoretical formula is introduced to calculate bending stress according to computed axial stress and the angle caused by relative movement between the main cable and the stiffening girder [36]. The Wyatt theoretical formula can only be applied to an object that is a round wire, which is not suitable for a set of strands such as the prototype bridge. Kondoh proposed that the bending stress in this kind of suspender at the joint was assumed to be 60% of the theoretical Wyatt formula based on the experimental results as Equation (11) [37]

(11) $$\sigma_{b}=1.2\tan\theta \cdot \sqrt{\sigma_{a}E}$$

where $\sigma_{a}$ is axial stress, *E* is the elasticity modulus of steel wire, and θ is the angle caused by the relative movement between the main cable and the stiffening girder.

Only the bending stress and axial stress of partial suspenders are shown due to layout constraints. The variation of the axial stress of short suspenders is small, whereas the length of short suspenders near the midspan is too small to release stress; the bending stress is greater than long suspenders. The influence of bending stress cannot be neglected in the analysis of suspender degradation. The settlement of flexible buckles considerably reduces the bending stress of suspenders but has minimal effect on the axial stress. The bending stress of short suspenders near suspender no. 26 (midspan) slightly decreases, whereas the long suspenders are almost unaffected. Thus, short suspenders nos. 21–26 are selected as analysis objects.

**Figure 13:**
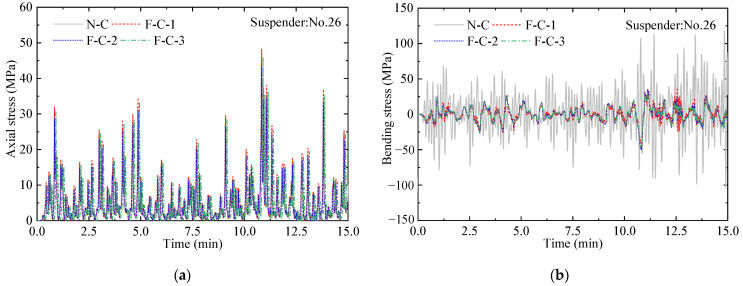
Time history of suspender stress (level 5). (**a**) Axial stress; (**b**) bending stress.

The traffic load is divided into different levels according to traffic density and then used for loading to calculate the structural dynamic response under traffic conditions. Figure 13 shows the suspender stress under the traffic flow at level 5. The time history of axial stress is consistent for different conditions, and the bending stress presents a remarkable difference in that the peak values are greatly reduced.

**Figure 14:**
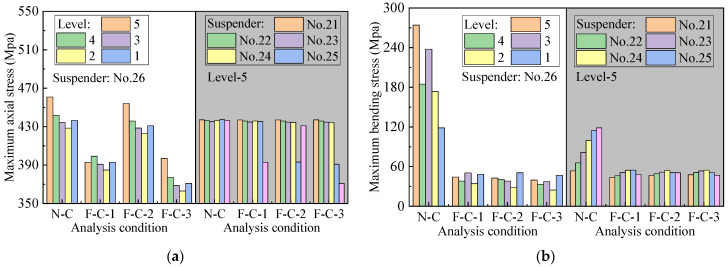
Comparison of peak values of stress. (**a**) Axial stress; (**b**) bending stress.

Figure 14 shows the peak values of bending stress and axial stress. The suspender stress response under different traffic flows is different, and the axial stress is slightly influenced by different traffic flows, whereas the bending stress is greatly reduced by flexible central buckles. The settlement of flexible central buckles has a certain influence on the axial stress and bending stress of short suspenders. The axial stress peak values vary under different conditions. F-C-1 has an improvement effect on suspender no. 26, whereas F-C-2 has an improvement effect on suspender no. 25; these results are related to the position of buckles. The settlement of buckles shares the axial stress of the suspender between inclined cables. In terms of bending stress, the flexible central buckle can remarkably reduce the peak value of short suspenders, but the weakening effect is not significantly improved with the increase in the number of buckles.

**Figure 15:**
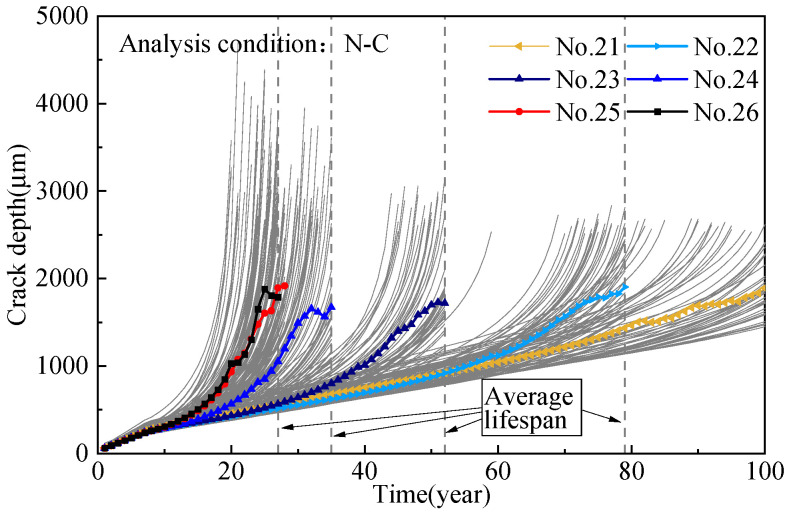
Development of average crack depth.

**Figure 16:**
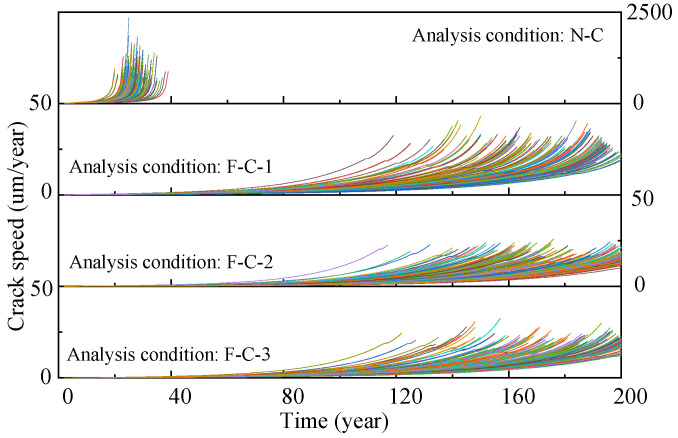
Crack speed of wire samples (suspender No. 26).

**Figure 17:**
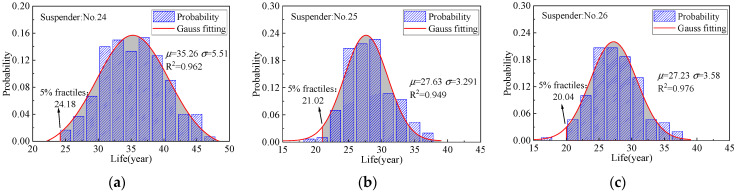
Distribution of steel wire life under the N-C condition. (**a**) Suspender no. 24; (**b**) suspender no. 25; (**c**) suspender no. 26.

The generation of pitting corrosion is a random process, and the maximum pitting depth directly affects the generation of cracks and fatigue life. In order to reflect the difference in steel wire life, the corrosion fatigue degradation of steel wire under different working conditions was simulated. A total of 150 samples for each analysis condition were sampled based on randomly generated maximum pitting coefficients, and then the transition from pitting corrosion to cracking and the crack development were calculated on the basis of the proposed predicting process. Figure 15 shows the crack development of the steel wire samples of suspender nos. 21–26. Under the N-C condition, the average crack development in the steel wire samples of suspender nos. 24–26 is remarkably faster than that of suspender nos. 21–23, satisfying the service requirements. Although the corrosion resistance of the aluminum alloy steel wire is better than that of the galvanized steel wire, once the steel wire coating is consumed, the crack growth rate caused by the substrate pitting pit mainly depends on the stress response of the suspender. The bending stress of the short suspender under the N-C condition is larger, but the steel wire fatigue life remains lower than the design life of the bridge. Figure 16 shows the crack speed of the wires of suspender no. 26; the settlement of flexible central buckles substantially improves their fatigue life, and the improvement effect is similar to that of the rigid central buckle [17]. However, the increase in the number of buckles does not considerably weaken the crack growth rate. The fatigue lives were fitted, and the results are shown in Figure 17; all of them obey a normal distribution. The mean values of the fatigue lives of suspender nos. 24, 25, and 26 have small differences because the length of these suspenders is close. The 5% fractiles are taken as the characteristic service life, in which the service life of the steel wire has a 95% assurance rate. The service life of the short suspender is 20.04, 21.02, and 24.18, respectively. All of them are lower than expected, but the service life can be significantly improved by increasing the length of the steel wire; that is, reducing the bending stress.

Table 4 shows the comparison of the equivalent stress amplitude and fatigue life of the steel wires of suspender nos. 24–26. With consideration of the responses under traffic flow of five levels, the fatigue life of the steel wire after setting buckles meets the service requirements, but the increase in the number of buckles has hardly improved the life of the suspender steel wire. The 95% confidence interval results of fatigue life are shown in Table 4. When the number of samples is sufficient, the confidence interval length is small. The length of the confidence intervals of N-C steel wire is less than 1.5 years, and those of F-C are all less than 8 years. The average life of steel wire tends to be stable, thus the sampling results are proven to be reliable. The setting of buckles can considerably improve the extreme value of bending stress and the equivalent stress replication. When two buckles are settled, the extreme value and the equivalent stress amplitude of the steel wire tend to be stable, and increasing the number of buckles is unnecessary. The equivalent stress amplitude of axial stress is unaffected, and the suspender stress of long-span suspension bridges is determined by the dead load.

## 6. Conclusions

The influence of a flexible central buckle on suspension bridge vibration was remarkable, but the control effect on short suspenders is still unknown. This study established the corrosion fatigue degradation model of high-strength steel wire based on traffic composition and explored the influence of flexible central buckles on the corrosion fatigue life of suspenders under traffic flow. To improve the consideration of traffic flow, the WIM data were processed according to traffic density and used to analyze the suspender response under traffic flow of different densities. The fatigue life of short suspenders without buckles and with different numbers of buckles was analyzed based on monitoring traffic data. The following conclusions were drawn:

1. The intensity of traffic flow greatly influences the stress response of suspenders. The bending stress of short suspenders is considerably greater than that of long suspenders. The setting of flexible central buckles can effectively reduce the peak value of bending stress, but when the number of central buckles exceeds two, the increase in number does not remarkably weaken the bending stress. In addition, the buckles can share the axial stress of the suspender between inclined cables, and the weakening effect is affected by the setting position.
2. According to numerical analysis results, the fatigue life of short suspender wires under traffic load is remarkably lower than that of the other suspenders due to large bending stress (about 27–35 years). The setting of buckles can effectively reduce the equivalent bending stress amplitude, but the equivalent axial stress amplitude does not remarkably decrease. The improved stress state of the short suspenders considerably extends the fatigue life of the steel wires under traffic flow (about 174–179 years); by contrast, the increase in the number of buckles has a minimal effect on steel wire life and extreme stress values.

The dynamic motion of the bridges is complex for diverse loads. Moreover, the fatigue behavior of short suspenders and the vibration control effect are influenced by other loads, such as wind, earthquakes, and other special conditions. The optimal design of flexible central buckles should be studied further.

## Figures and Tables

**Table 1:** Comparison of stiffening girder vertical deflection under static test.

	L/8	3L/8	L/2	5L/8	7L/8
Measured Value (mm)	175	606	1093	615	180
FE model (mm)	181	624	1120	630	185
Error (%)	0.03	0.03	−2.02	0.02	0.03

**Table 2:** Parameters of steel wire samples.

	Nominal Diameter (mm)	Tensile Strength (MPa)	Yield Strength (MPa)	Modulus of Elasticity (GPa)	Coating Quality (g/cm^2^)	Coating Depth (μm)
Galfan steel wire	5.25	1926	1775	2.08$\times 10^{5}$	337	31.05

**Table 3:** Analysis conditions of the FE model.

Condition	Description	Schematic
N-C	No central buckle	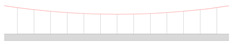
F-C-1	Single flexible central buckle	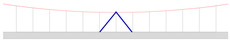
F-C-2	Double flexible central buckles	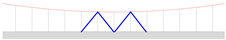
F-C-3	Three flexible central buckles	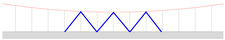

**Table 4:** Fatigue life of suspender wire under different analysis conditions.

Suspender Number (Length)	Analysis Condition	Maximum Bending Stress (MPa)	Equivalent Bending Stress (MPa)	Equivalent Axial Stress (MPa)	Fatigue Life (Year)		
					*μ*	*σ*	Confidence Intervals (95% CI)
24 (2.75 m)	N-C	208.31	37.30	10.23	35.2	5.51	(34.3,36.1)
	F-C-1	70.52	14.19	9.60	179.3	19.31	(176.2,182.4)
	F-C-2	54.14	11.38	9.51	178.5	19.54	(175.4,181.6)
	F-C-3	54.67	12.46	9.45	181.3	19.59	(178.2,184.4)
25 (2.45 m)	N-C	238.91	44.32	9.63	27.6	3.29	(27.1,28.1)
	F-C-1	77.16	15.68	10.12	177.8	19.32	(174.7,180.9)
	F-C-2	50.81	11.62	10.33	177.9	20.39	(174.6,181.2)
	F-C-3	50.79	11.81	9.38	181.1	20.10	(177.9,184.3)
26 (2.41 m)	N-C	274.10	46.66	10.03	27.2	3.58	(26.6,27.8)
	F-C-1	72.17	14.38	10.17	174.2	19.49	(171.1,177.3)
	F-C-2	50.68	11.93	8.98	178.1	21.35	(174.7,181.5)
	F-C-3	46.85	12.07	9.30	179.4	20.60	(176.1,182.7)

## Data Availability

Some or all data, models, or codes that support the findings of this study are available from the corresponding author upon reasonable request.
